# Implementation of an Infection Prevention Care Bundle for Peripheral Intravenous Catheters (PIVCs): A Quality Improvement Study to Enhance PIVC Quality and Reduce Complications

**DOI:** 10.3390/nursrep15110379

**Published:** 2025-10-24

**Authors:** Kristine Amble, Ingun Børve Skjelbreid, Geir Egil Eide, Susann Muri, Lise Husby Høvik, Marit Hegg Reime

**Affiliations:** 1 Haraldsplass Deaconess Hospital, Ulriksdal 8, 5009 Bergen, Norway; kristine.amble@haraldsplass.no (K.A.); susann.helen.tarberg.muri@haraldsplass.no (S.M.); 2 Haukeland University Hospital, Jonas Lies Vei 65, 5021 Bergen, Norway; ingun.borve.skjelbreid@helse-bergen.no; 3 Department of Health and Caring Sciences, Faculty of Health and Social Sciences, Western Norway University of Applied Sciences, Inndalsveien 28, 5063 Bergen, Norway; geir.egil.eide@haukeland.no; 4 Clinic of Anaesthesia and Intensive Care, St. Olav’s Hospital, Trondheim University Hospital, Prinsesse Kristinas Gate 3, 7030 Trondheim, Norway; lise.husby.hovik@stolav.no; 5 Department of Postgraduate Studies, Lovisenberg Diaconal University College, Lovisenberggata 15B, 0456 Oslo, Norway

**Keywords:** bloodstream infections, care bundle, nursing, peripheral intravenous catheter, phlebitis, PIVC-miniQ, predictors, quality improvement

## Abstract

**Background/Objectives:** Peripheral intravenous catheters are commonly employed to administer intravenous therapy to hospitalized patients. However, their use can result in complications, with phlebitis occurring in approximately 11% of cases and bloodstream infections in about 0.18%. This study aimed to enhance PIVC management in a local hospital by implementing a comprehensive care bundle to mitigate these complications. **Methods:** This quality improvement study involved the collection of data from 1330 PIVCs in adult patients, both prior to and following the implementation of the intervention. Data collection occurred between June 2022 and November 2023, employing the validated Peripheral Intravenous Catheter-Mini Questionnaire (PIVC-miniQ). This instrument comprises 16 observation points that assess phlebitis-related signs and symptoms, the integrity of PIVC dressings and IV connections, and the adequacy of documentation. **Results:** The prevalence of phlebitis decreased from 15.1% at baseline to 9.4% post-intervention (*p* = 0.018). Significant predictors of phlebitis included the intervention, ward, gender, and PIVC gauge. Improvements were also noted in PIVC dressing and IV connection practices, as well as documentation standards (*p* < 0.001). Closed integrated PIVCs outperformed ported PIVCs in the PIVC-miniQ scores after the intervention (*p* < 0.001). A statistically significant difference was observed in the mean PIVC-miniQ sum score post-intervention compared to baseline (*p* < 0.001). **Conclusions:** This study indicates that implementing a care bundle can enhance the quality of PIVCs and reduce the prevalence of phlebitis. Further high-quality research is needed to identify the most effective care bundles for preventing PIVC-related complications.

## 1. Introduction

### Problem Description

The placement of peripheral intravenous catheters (PIVCs) is the most frequently performed procedure for administering intravenous (IV) fluids or medical treatment to hospitalized patients [1,2]. There are various types of PIVCs, where one is identified as a closed integrated system [3] featuring large wings and a preassembled extension [4], in contrast to the traditional non-integrated and open system PIVC [3], referred to as ported PIVCs. As many as 70% of hospitalized patients receive a PIVC [5]. However, the use of PIVCs can result in various complications, including infiltration, occlusion, dislocation, phlebitis [6], bloodstream infections [7], and catheter failure. The latter refers to the unexpected removal of the catheter due to malfunction or other related complications [3]. Many patients receive PIVCs without a clear indication, with studies showing some to be unnecessary or lacking IV treatment orders [8,9,10,11,12]. To reduce the risk of complications, several recommendations have been established [13]. Healthcare personnel should closely monitor PIVCs to determine the need for dressing changes, check for irregularities in the IV set, and ensure proper antiseptic preparation of the skin prior to insertion and during dressing changes [13]. Additionally, healthcare practitioners should use an aseptic non-touch technique when handling the PIVC and its connections and promptly remove any PIVCs that are non-functional or unnecessary [13].

The insertion of a PIVC carries the risk of phlebitis [14]. A systematic review and meta-analysis found that 11% of catheters were affected by phlebitis [14]. Phlebitis can occur due to chemical, mechanical, or bacterial causes [13] and can be defined as inflammation of the vein, which gives symptoms of pain, tenderness, induration, and/or erythema along the vein [15]. Although phlebitis itself is not an infection, it can lead to one [15]. When phlebitis is accompanied by the development of a blood clot, it is referred to as thrombophlebitis [16]. It is recommended to use the smallest possible PIVCs, as larger catheters tend to adhere more to the vessel wall and, therefore, increase the risk of phlebitis [17]. If any signs and symptoms of phlebitis are detected, the PIVC should be removed [13].

In addition to phlebitis, PIVCs can lead to bloodstream infections (BSIs) when pathogenic bacteria enter the bloodstream, leading to severe illness and potential death [18]. A systematic review reported that out of 85,063 PIVCs, the rate of PIVC-related BSIs was 0.18% [1]. PIVC-BSIs can result in extended hospital stays, prolonged antibiotic treatment, intensive care, and potentially even death [19]. The most common microorganism in PIVC-BSIs is *Staphylococcus aureus* (*S. aureus*) [11,20,21], with high mortality rates [19,22]. In accordance to Trinh et al. [23], a probable PIVC-BSI is based on the fulfilment of the following three criteria: (1) *S. aureus* isolated from blood cultures obtained during hospitalization and after the insertion of a PIVC; (2) no other identifiable source of infection is present; (3) clinical signs of infection at the catheter insertion site (e.g., phlebitis, redness, tenderness) are evident. PIVCs are typically used for short-term treatment and have a reduced chance of complications compared to central intravenous catheters [24]. However, the evaluation of the effectiveness of preventive measures for bloodstream infections in Norway is currently hindered by the lack of a national surveillance system dedicated to this issue [24]. Furthermore, infections associated with PIVCs may remain unrecognized due to their relatively short indwelling times and the brief duration of hospital stays [25]. This frequently results in the catheter being removed before the PIVC-BSI is identified, suggesting a potential underdiagnosis and underreporting of these infections [19]. Given that PIVCs are among the most commonly utilized invasive devices in clinical settings, the worldwide impact of PIVC-BSIs is likely to be significant [5]. Consequently, the purpose of this longitudinal quality improvement study was to enhance PIVC management processes, achieve measurable improvements, and systematically document the results to inform future quality improvement initiatives.

## 2. Background

### 2.1. Healthcare-Associated Infections, Patient Safety, and Infection Prevention

Healthcare-associated infections (HAIs) are infections that patients acquire while receiving treatment at a healthcare facility, infections that were not present upon admission [26,27]. BSIs are considered to be one of the most preventable of all HAIs [5]. HAIs represent a global challenge, resulting in prolonged hospital stays, patient suffering, increased antibiotic resistance, and significant economic costs [27]. In the European Union, it is estimated that over 3.5 million instances of HAI occur annually, resulting in more than 90,000 deaths [26]. Notably, approximately 50% of these HAIs could be prevented through effective interventions, underscoring the critical importance of prevention efforts in reducing HAI incidence [26]. In 2024, a total of 522,989 patients were admitted to Norwegian hospitals [28]. A point prevalence survey conducted in May 2024 on hospitalized patients revealed that 2.7% had HAIs, and 0.3% had BSIs [29].

There is a scarcity of research on the prevention of infections related to PIVCs, and as a result, guidelines are primarily based on studies related to central intravenous catheters [24]. A longitudinal study conducted between 2007 and 2019 on catheter-related BSIs revealed that PIVC-BSIs were increasing in medical wards, underscoring the need for intervention studies aimed at preventing infections in PIVCs [30]. Contrary, a multicenter intervention study did not find a significant reduction in PIVC-related infections after implementing an evidence-based care bundle and conducting educational sessions for healthcare personnel, suggesting that healthcare personnel might not perceive PIVCs as a significant patient safety risk [21]. Furthermore, evidence indicates considerable variation in PIVC management practices across different care settings internationally [9], and nursing education tends to emphasize insertion techniques over the maintenance of PIVCs [10]. Additionally, nurses often rely on personal judgment rather than updated clinical guidelines when it comes to PIVC practices [10]. The World Health Organization (WHO) advocates for appropriate training for healthcare personnel responsible for the insertion of intravascular catheters [5]. To use PIVCs in a way that effectively prevents phlebitis, evidence-based training is essential to uphold high standards of patient care [17].

### 2.2. Care Bundles

Through comprehensive searches in the Cinahl, Embase, and Medline databases, we identified studies examining preventative interventions for phlebitis and bloodstream infections associated with the use of PIVCs [17,31,32,33,34,35,36]. Care bundles consist of different evidence-based interventions targeting the same patient group or healthcare area and typically yield better outcomes when implemented collectively rather than individually [5,37,38]. One study found that the implementation of PIVC care bundles can enhance patient safety by reducing PIVC-BSI rates [33]. However, a systematic review revealed that the evidence regarding the effectiveness of PIVC care bundles in reducing BSIs is still inconclusive, underscoring the need for further research to identify and standardize the most effective components of these care bundles [35]. “The Peripheral Intravenous Catheter-Mini Questionnaire” (PIVC-miniQ) is a screening tool designed to assess the prevalence of deviations in the PIVCs and evaluate local improvement interventions aimed at enhancing the quality of PIVC management in hospital wards [39]. Several studies have utilized some or all of the items from the PIVC-miniQ [39,40,41,42,43,44]. However, none of these studies have employed the tool in a longitudinal research context, nor have they compared PIVC quality before and after interventions [39,40,41,42,43,44].

### 2.3. Specific Aims

The rationale for conducting this study arose from the identification of BSIs related to PIVCs in our local hospital.

Additionally, there was a notable lack of documentation regarding the daily observation and maintenance of PIVCs, as well as a shortage of comprehensive data on complications and infections associated with these catheters. In 2021, the Norwegian Institute for Public Health (NIPH) published updated guidelines for the usage of intravascular catheters [24], prompting the hospital to revise its internal guidelines for the insertion and maintenance of PIVCs to comply with the new standards. Consequently, the aim of this study was to implement a longitudinal quality improvement initiative using the PIVC-miniQ quality assessment tool [39] to evaluate a local improvement care bundle designed to enhance the insertion, maintenance, removal, and documentation of PIVCs, ultimately aiming to prevent PIVC-related complications, such as phlebitis and BSIs.

### 2.4. Research Questions

1. Prevalence of Phlebitis: What is the prevalence of phlebitis among patients with PIVCs, and does the prevalence decrease after the implementation of the care bundle?
2. Predictive Factors for Phlebitis: Which factors are associated with and can predict phlebitis?
3. Improvement in PIVC Dressing, IV Connection, and Documentation Quality: Has there been an improvement in the quality of the documentation, as well as the quality of the PIVC dressing and the IV connection, following the implementation of the care bundle?
4. Comparison of PIVC-miniQ Scores: Is there a difference in the PIVC-miniQ sum score when comparing closed integrated PIVCs to ported PIVCs?
5. Change in PIVC-miniQ Sum Score: Does the PIVC-miniQ sum score decrease after the implementation of the care bundle?

## 3. Materials and Methods

### 3.1. Design

The study was designed as a quality improvement initiative that included baseline and post-intervention measurements of patients with PIVCs [45] and is reported in accordance with the recommendations outlined in the Standards for Quality Improvement Reporting Excellence 2.0 (SQUIRE 2.0) guidelines [46].

### 3.2. Quality Improvement

**Figure 1:**
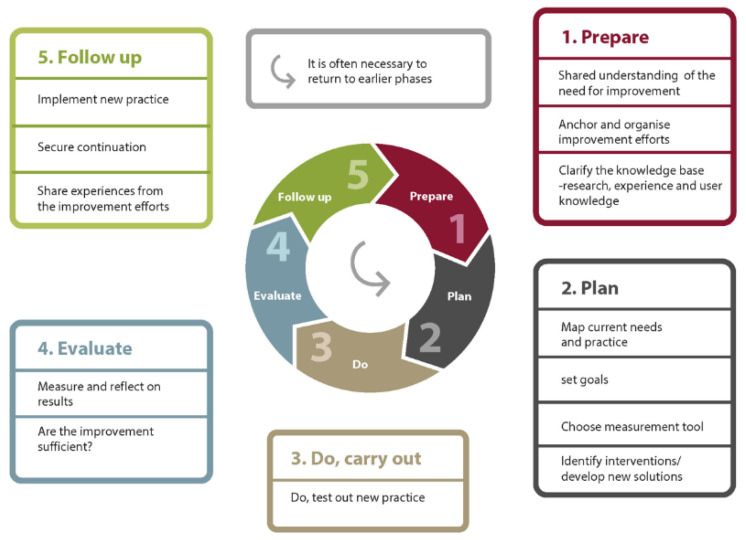
Quality Improvement Model reprinted from the Norwegian Institute for Public Health with permission [47].

Within healthcare, quality improvement involves systematically and persistently working towards measurable enhancements [47] that lead to sustainable positive changes for patients, the healthcare system, and professional development [47,48]. For this project, we utilized the Quality Improvement Model as our framework, as illustrated in Figure 1 [47]. This model is rooted in research and practical experience, providing the necessary methods for enhancing services [47]. The model’s circular design symbolizes the continuous nature of improvement efforts, highlighting the importance of ongoing adjustments to sustain and enhance results [47].

### 3.3. Sample

The quality improvement study involved patients ≥ 18 years old who were admitted to five inpatient wards. Convenience sampling was employed to target patients who had at least one PIVC in place [45]. Patients who were not present during the observation, those who were terminally ill, and individuals deemed inappropriate for observation by the nursing staff due to their medical condition were excluded.

### 3.4. Setting

This quality improvement study was conducted at a large local hospital in Norway, encompassing five inpatient medical and surgical wards. The hospital serves a population of 145,000 residents and has a total of 142 beds. Additionally, it offers surgical services across various specialties, performing 6216 surgeries in 2024. In recent years, the hospital has undergone structural changes and implemented various improvements, resulting in the hospital receiving quality awards. These initiatives may have made the organization more open to improvement approaches [49] and helped foster a culture conducive to further advancements [50]. However, time constraints and the multitude of tasks within a large hospital setting may pose significant barriers to implementing new interventions [50].

### 3.5. Care Bundle Interventions

During the study period from June 2022 to November 2023, several key interventions were implemented to improve PIVC care and ensure compliance with established guidelines. Firstly, the hospital’s PIVC guideline was updated in accordance with the NIPH guideline [24]. Prior to the intervention, the hospital utilized two types of PIVCs: the previously mentioned ported PIVC and a straight PIVC, referred to as a non-ported PIVC. The non-ported PIVC is designed to minimize blood exposure during insertion [51]. This PIVC is rarely used in the hospital and is reserved for ultrasound-guided insertion. We subsequently implemented a closed integrated PIVC system in the inpatient wards, resulting in a total of three PIVC types being used in the hospital. Closed integrated PIVC systems are associated with fewer catheter failures when compared to non-integrated systems [52]. The decision to adopt closed integrated PIVCs was supported by studies demonstrating a reduction in complications [53,54,55,56,57,58].

In September 2022, over a two-week period, all employees involved in the insertion and care of PIVCs participated in a comprehensive 40 min educational session. This training encompassed information on the infection prevention bundle and provided practical instructions on handling the closed integrated PIVC. Pre-intervention data and adverse events related to PIVCs in our hospital were presented to emphasize the importance of the study and to engage and motivate the employees [59]. Other topics aligning with the NIPH guideline [24] included the importance of hand hygiene and aseptic technique when handling PIVCs. Additionally, interventions such as disinfection of skin and IV connections, proper flushing techniques, and appropriate bandaging were also emphasized in the educational session [24]. We included daily observation of the PIVC insertion site and dressing, along with a daily assessment of its indication, and removal of the PIVC when it was no longer needed or in cases of signs of infection [24]. Furthermore, a shift was made from routinely replacing the PIVC every 96 h to replacing it based on clinical indications [24]. In addition, we emphasized the importance of documenting these interventions, as mandated by healthcare legislation [60]. Other essential topics addressed included the selection of catheter size (gauge) and the identification of optimal insertion sites [24]. To reinforce these practices, an instructional video was made available through the hospital’s learning management system. Six months later, the educational sessions were repeated and subsequently integrated into training courses for new employees and nursing students [50].

**Figure 2:**
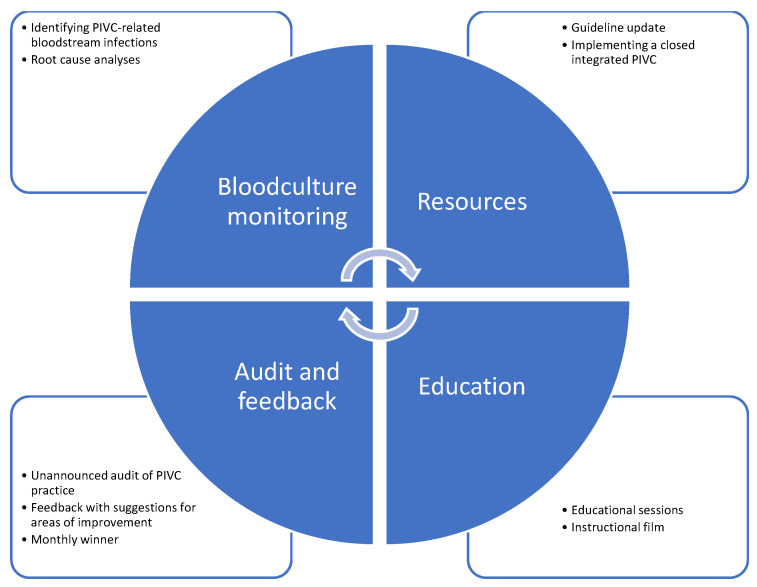
Care bundle interventions.

Additionally, unannounced audit and feedback sessions to evaluate PIVC practices and assess the local improvement of the interventions using the PIVC-miniQ tool [39] were conducted monthly from November 2022 to November 2023 [24,49,59]. Baseline measurements were obtained in June and August 2022. Each ward received feedback on its performance, accompanied by suggestions for improvements. To foster a culture of continuous improvement, a monthly winner was announced [50]. Furthermore, we initiated blood culture monitoring as part of the study due to the absence of data on PIVC-related BSIs [24]. Consequently, we did not have information on BSIs prior to the intervention. Root cause analyses were conducted whenever a PIVC-related BSI was identified. An overview of the care bundle interventions implemented in the study is presented in Figure 2.

The team involved in the project consisted of eight members: one representative from each of the five wards’ management teams, one nurse anesthetist, and two infection control nurses who led the study. All team members were experienced nurses, were stakeholders in their respective wards, and worked during daytime hours, facilitating regular meetings and the implementation of interventions [49,61,62]. Additionally, the quality improvement study included a steering committee made up of the leaders of the wards and the infection control physician [49]. The directors of the medical and surgical clinics also served as sponsors for the initiative.

### 3.6. Data Collection

The validated audit tool PIVC-miniQ [39] was utilized with the developers’ permission to collect data. A total of 10 experienced nurses participated in the data collection after completing a two-hour training session on scoring the relevant variables. Data collection occurred once a month on weekdays between 9 a.m. and 3 p.m. The nurses worked in pairs across the five wards, visiting all patients to observe and determine whether they had a PIVC in place. One nurse assessed the patient’s PIVC, while the other recorded the observations using a paper-based PIVC-miniQ. If the patient had multiple PIVCs, each one was examined and documented on a separate form. In cases where signs of infection were unclear, both nurses observed the PIVC to reach a consensus. Given that documentation of PIVC insertion date may often be lacking in the patient’s chart [10,39,42,44], this observational method for identifying patients with PIVC was deemed the most reliable. A unique patient code was assigned to each form, which was linked to a printout of the patient list. Documentation in the patient’s electronic chart was reviewed either at the end of the shift or in the following days. The patient list, along with their corresponding codes, was securely saved and subsequently shredded after being recorded in Microsoft Excel for Microsoft 365 MSO version 2408 [63]. This process ensured that the data remained anonymized.

### 3.7. Included Audit Tool and Variables

The PIVC-miniQ tool can reliably measure the overall quality of PIVC care in point-prevalence audits and can be utilized to improve patient safety [39]. It has been validated in both medical and surgical departments across two Norwegian hospitals [39,64]. Two studies have examined the instrument’s reliability by assessing inter-rater reliability using the intraclass correlation coefficient (ICC) [39,43]. The ICC between raters was, respectively, 0.604 [39] and 0.48 [43], confirming that the PIVC-miniQ is a reliable instrument [39,43,65].

The PIVC-miniQ consists of four domains, encompassing a total of 16 nominal-level variables. Each deviation from established standards results in the allocation of one point [39]. Points are summarized, with an optimal score of zero indicating high quality in PIVC management [39]. The “Phlebitis-related signs and symptoms” domain includes nine items, examining the insertion site for indicators of infection [39]. The “PIVC dressing and IV connection” domain consists of five items focusing on deviations related to the PIVC dressing or IV connection [39]. The “Documentation” domain comprises one item that assesses the adequacy of PIVC documentation in the patient’s medical record, while the “Indication” domain contains a single item evaluating whether there is an appropriate indication for the use of the PIVC [39]. Additionally, demographic information was recorded for each patient and each PIVC; however, these details are not included in the total score [39].

In accordance with the study by Helland et al. [44], several adjustments were made regarding the domains and their associated items during the data analysis. Firstly, the item “Partial/complete dislodgement” was transferred from the “Phlebitis-related signs and symptoms” domain to the “PIVC dressing and IV connection” domain, as it does not pertain to phlebitis assessment [40,44]. Additionally, the “Documentation” and “Indication” domains were merged [44] to ensure that a valid indication is documented in the patient’s medical record. The item “Insertion date not documented on PIVC dressing” was moved to the “Documentation” domain [44]. In this quality improvement study, an additional documentation item, “Daily documentation of observation, care, and indication assessment in chart is missing”, was introduced, raising the total number of items in the combined “Documentation” domain to four, while increasing the overall PIVC-miniQ score to 17 points (Supplementary Materials). This item was added due to the necessity for daily observation of PIVCs, particularly as we transitioned our guidelines from routine replacement of PIVCs to replacement based on clinical indications [24].

### 3.8. Statistical Analyses

Descriptive statistics included mean and standard deviation (SD) for continuous variables and frequencies (n) and proportions (%) for categorical variables [45]. We compared pre-intervention scores to post-intervention scores using the independent *t*-test for continuous data and chi-square tests for categorical data [45]. To assess time trends in the prevalence of phlebitis, we employed the chi-square test for linear-by-linear association. A *p*-value ≤ 0.05 was considered statistically significant [45]. PIVC-miniQ sum scores were summarized for all PIVCs before and after the intervention, with the distributions presented in histograms.

We employed logistic regression analysis to investigate potential predictors associated with phlebitis [45]. Phlebitis was defined as one or more deviations within the “Phlebitis-related signs and symptoms” domain of the PIVC-miniQ [44]. Firstly, each predictor variable was analyzed separately, and unadjusted ORs with 95% CIs and *p*-values (likelihood ratio test) are reported. Secondly, a fully adjusted multiple logistic regression model with all predictors was estimated, and fully adjusted ORs, CIs, and *p*-values are reported. Finally, starting with the fully adjusted model, a backward stepwise selection process was performed, removing the least significant variable at each step, and ending when only variables with *p* ≤ 0.05 were retained in the model. This final model is reported with ORs, CIs, and *p*-values. Collinearity between predictors in the multiple regression analyses was assessed using the variance inflation factor (VIF). A VIF > 5 was considered to indicate a potential collinearity problem.

In addition to examining phlebitis, we performed logistic regression analyses to study two other domains of the PIVC-miniQ: “PIVC dressing and IV connection” and “Documentation”. These analyses aimed to determine the prevalence of deviations (sum score ≥ 1) within these domains, assess differences, and estimate the risk of deviations before and after the intervention [45]. We also performed a Kruskal–Wallis one-way analysis of variance comparing the PIVC-miniQ sum score among different types of PIVC post-intervention. All statistical analyses were carried out using IBM SPSS Statistics for Windows, Version 30.0.0.0 [66].

### 3.9. Ethical Considerations

This quality improvement study was conducted in accordance with the principles of the Declaration of Helsinki [67]. Approval for the study was granted by the hospital’s data protection officer (case 3354). The Regional Committee for Medical and Health Research Ethics (REK) classified the study as a quality improvement initiative, determining that it fell outside the scope of the Health Research Act and therefore did not require further ethical approval (no. 501676). All collected data were stored in compliance with the hospital’s guidelines on a secure server.

## 4. Results

During the study period, a total of 1330 PIVCs across five hospital wards were screened. The wards included one surgical ward, two medical wards, one intensive care unit, and one observation unit. Table 1 presents demographic variables for patients and PIVCs.

### 4.1. Prevalence of Phlebitis

Table 2 shows that pain was the most frequently documented symptom at the insertion site, and both pain and induration improved significantly post-intervention. Swelling and redness also improved, but the results were not statistically significant. Table 3 shows that the prevalence of phlebitis decreased significantly, from 15.1% before the intervention to 9.4% afterward. Additionally, the risk of phlebitis was 61% higher pre-intervention compared to post-intervention.

**Figure 3:**
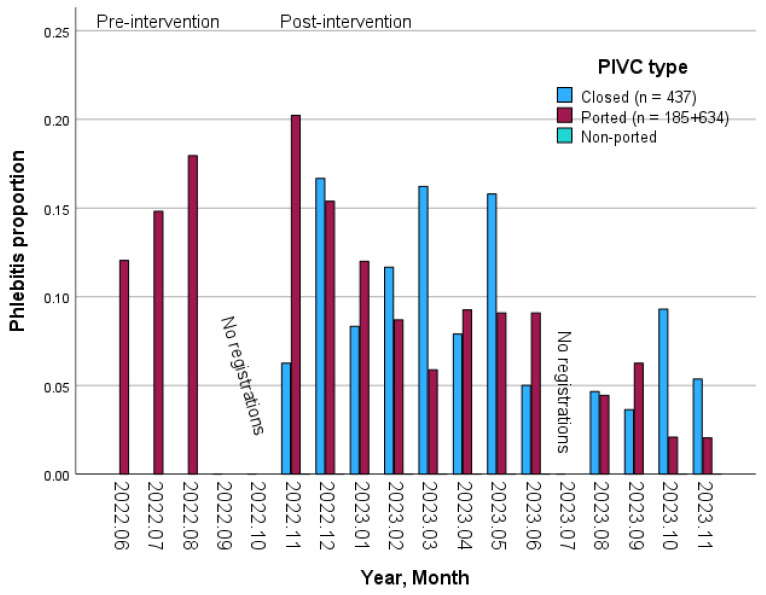
Monthly prevalence of phlebitis during the pre- and post-intervention period according to the type of PIVC. Phlebitis was defined as having the “Phlebitis-related signs and symptoms” domain of the PIVC-miniQ sum score ≥ 1. The non-ported PIVCs were too few to appear in the figure (n = 14). The post-intervention period test of overall linear trend was *p* < 0.001 (ported PIVCs: *p* < 0.001; closed integrated PIVCs: *p* = 0.092).

Figure 3 illustrates the monthly prevalence of phlebitis during the pre- and post-intervention periods, categorized by the type of PIVC. The data reveals an overall linear trend (*p* < 0.001).

### 4.2. Predictors of Phlebitis

Table 4 illustrates the results from the logistic regression analysis of phlebitis, including 1330 PIVCs, and the final model included four predictors. The intervention reduced the odds for phlebitis by 47% (adjusted OR = 0.53), being a female increased the odds of phlebitis by 75% (adjusted OR = 1.75), and the PIVC gauge had an adjusted OR of 5.42 per mm. Finally, the risk of phlebitis varied across the type of ward, the odds being 75% higher in the observational unit, 65% higher in the surgical ward, and 56% higher in medical ward 2, all compared to medical ward 1. In the ICU, there were no cases of phlebitis. Multicollinearity did not seem to be a problem as all variance inflation factors were small (Table 4).

### 4.3. Quality of the PIVC Dressing and IV Connection and the Documentation

Table 2 indicates that the most frequently observed problem in the “PIVC dressing and IV connection” domain was “soiled with blood or fluids”, both at baseline and post-intervention. Notably, this item showed significant improvement, decreasing from 43.8% to 25.2% post-intervention. Additionally, the items “Blood in line” and “Loose or lifting dressing edges” also demonstrated significant improvement. Furthermore, Table 3 reveals that before the intervention, there was a 52% higher risk of experiencing one or more deviations in the “PIVC dressing and IV-connection” domain. Table 2 shows that the quality of the documentation significantly improved from baseline to post-intervention in three out of four items, namely “Insertion date not documented on PIVC dressing”, “Documentation of indication in chart is missing,” and “Daily documentation of observation, care, and indication assessment in chart is missing”. The logistic regression analysis for the PIVC-miniQ domain “Documentation” indicates that the risk of deviation was 27% higher before the intervention than after (Table 3).

### 4.4. PIVC Type and PIVC-miniQ Sum Score

**Figure 4:**
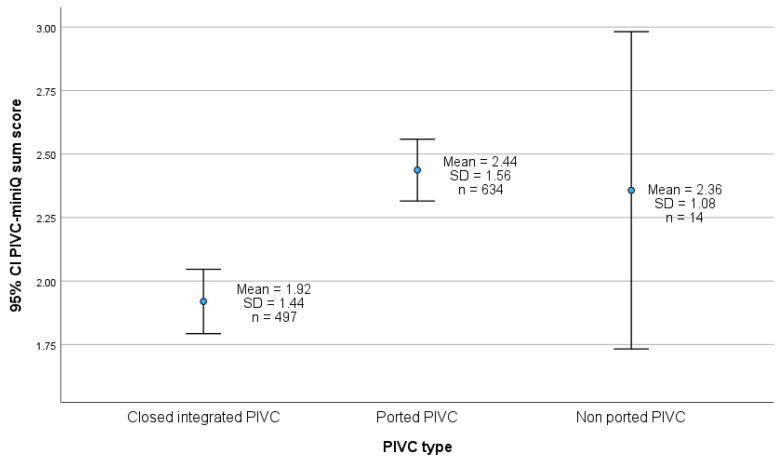
PIVC type and mean PIVC-miniQ sum score for 1145 PIVCs after the intervention. SD = standard deviation. F-test *p*-value < 0.001 from one-way analysis of variance.

Figure 4 illustrates that the PIVC quality is better using the closed integrated PIVCs compared to the ported PIVCs. The mean PIVC sum score for closed integrated PIVC is 1.92 (SD 1.44) and 2.44 (SD 1.56) for ported PIVC, and the difference between the two groups is statistically significant (*p* < 0.001).

### 4.5. PIVC-miniQ Sum Score

**Figure 5:**
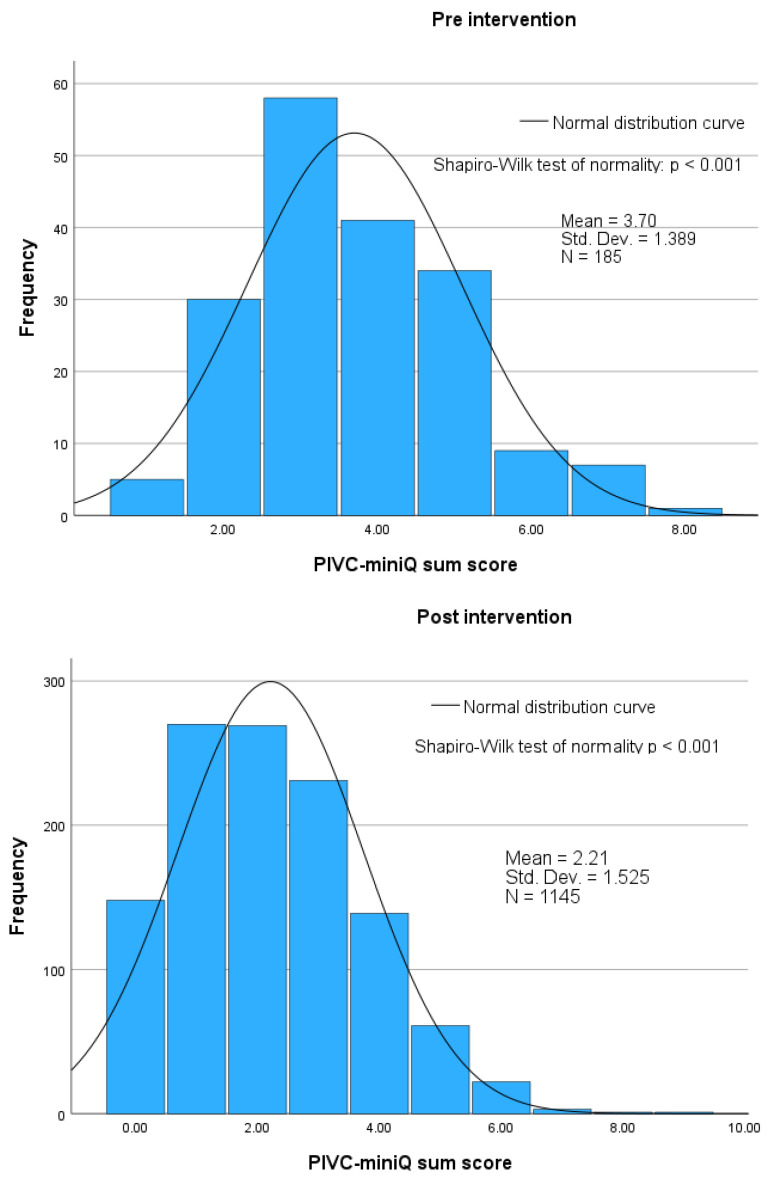
The distribution for the PIVC-miniQ sum score for 185 PIVCs pre-intervention and the PIVC-miniQ sum score for 1145 PIVCs post-intervention, and comparisons with the normal distribution.

**Figure 6:**
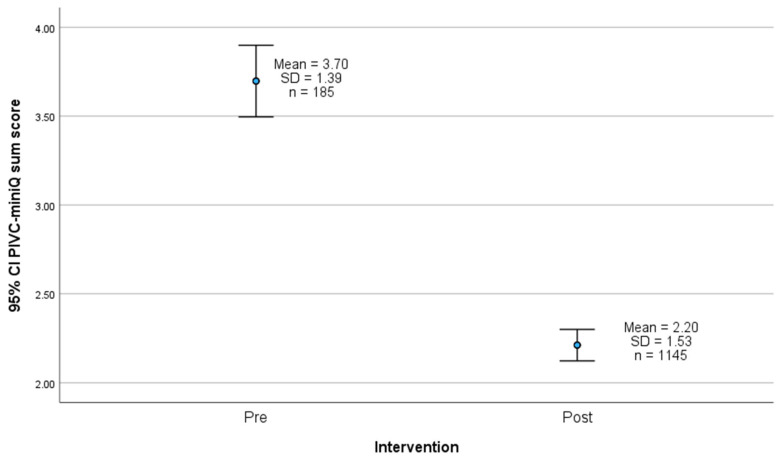
Mean PIVC-miniQ sum score before and after the intervention with 95% confidence intervals (CI). SD = standard deviation. T-test *p*-value < 0.001.

The distribution of PIVC sum scores before and after the intervention is illustrated in Figure 5, showing a mean sum score of 3.70 (SD 1.39) pre-intervention and 2.20 (SD 1.53) post-intervention. Figure 6 highlights the difference between the pre- and post-intervention means, which is 1.45 (95% CI: 1.25–1.72), indicating a statistically significant difference (*p* < 0.001).

## 5. Discussion

Our aim was to conduct a longitudinal quality improvement study to assess whether a care bundle intervention could reduce complications related to PIVCs. The intervention led to a statistically significant reduction in local phlebitis rates. Additionally, we identified several predictors of phlebitis, including the intervention itself, hospital ward, gender, and PIVC gauge size. Furthermore, there were local improvements in the quality of PIVCs regarding several phlebitis-related signs and symptoms, PIVC dressings, IV connections, and documentation practices.

### 5.1. Prevalence of Signs and Symptoms of Phlebitis

The most common signs and symptoms associated with phlebitis, both before and after the intervention, were pain and redness. This finding aligns with previous studies that have utilized the PIVC-miniQ tool [39,43,44]. However, none of these studies implemented interventions; thus, our study contributes valuable knowledge to the research field by demonstrating a significant reduction in the most frequently documented symptom, pain, and a significant decrease in the prevalence of phlebitis during the intervention. Besides, there was also a reduction in redness, although it was not statistically significant. Upon examining the monthly linear trend in the prevalence of phlebitis by type of PIVC following the intervention, we observed a statistically significant decrease in the overall data as well as for ported PIVCs. However, the closed integrated PIVCs did not demonstrate a significant trend.

In a systematic review, Ray-Barruel et al. [2] identified 71 phlebitis assessment scales that had not been properly validated, each incorporating various phlebitis-related signs. There is currently no widely agreed-upon definition for the measurement of phlebitis [68,69], and the incidence of phlebitis is also reported differently [2,69]. This lack of standardization complicates the comparison of results across different studies [2,68]. Our study defines phlebitis as the presence of one or more signs of phlebitis. This aligns with the definition used by Helland et al. [44], who also employed the PIVC-miniQ tool, facilitating easier comparison of our results. Moreover, PIVC-miniQ is a validated and reliable instrument [39,43], making it suitable for comparing research findings across national borders. Nevertheless, there is a pressing need for a standardized agreement on phlebitis measurement to enhance reporting accuracy and improve the comparability of results across studies [2,69,70].

### 5.2. Predictors of Phlebitis

Our study identified four predictors of phlebitis: the size of the peripheral intravenous catheter (PIVC gauge), gender, ward, and intervention. Understanding these risk factors for phlebitis can assist healthcare professionals in mitigating risks by developing targeted interventions to prevent complications associated with the use of PIVCs [71]. Other studies have identified additional predictors of phlebitis, including age, presence of chronic disease, drug and blood administration through the same vein, cannula gauge size, improper PIVC dressings, PIVC dwelling time [72], the quantity of missed nursing care [73], vein quality, use of contrast agent, hand hygiene practices, and nursing experience [74].

Regarding the size of the peripheral intravenous catheter, previous research has similarly shown that larger PIVCs are associated with an increased incidence of phlebitis [17,75,76]. It is thus recommended to use the smallest possible PIVCs to minimize this risk [17]. Furthermore, we observed an increase in the insertion of PIVCs in joint areas, such as the antecubital fossa and wrist, from baseline to post-intervention, along with a rise in the use of larger PIVCs (i.e., 18 gauge). The movement of the PIVC is more frequent when it is placed in joint areas, which can potentially cause mechanical phlebitis [17]. Therefore, our findings indicate a need for improvements in PIVC practices in this regard. Although our results were not statistically significant, it is concerning that evidence-based guidelines regarding PIVC size and placement were not adhered to, even after educational sessions conducted prior to the intervention. Nonetheless, our findings align with those of a large international study, which reported that 69% of PIVCs were inserted in the hand, the antecubital veins, and the wrist—areas not recommended for PIVC placement [8]. The veins in the forearm are recommended as optimal sites for PIVC insertion in adults due to their wide surface area, which facilitates secure dressing and stabilization of the PIVCs [8]. However, placing PIVCs in the wrist or antecubital fossa increases the risk of colonization and local infection [77]. Similarly, Høvik et al. [39] found that many of the PIVCs were positioned close to joint areas and were larger than recommended. They also observed that PIVCs were not removed despite signs of redness and reports of pain at the insertion site [39]. Other studies have also demonstrated that the management of PIVCs in healthcare settings often deviates from established clinical guidelines [10,78] and that nurses’ knowledge of best practices is insufficient [10,78,79]. Our study did not investigate the reasons why nurses selected larger PIVCs and positioned them in joint areas, which would be an interesting area for exploration in qualitative research.

We found that being a woman is a predictor of phlebitis. This supports findings from a systematic review that reported higher rates of complications related to PIVCs in women [17]. Additionally, a secondary analysis of risks associated with PIVCs, along with a multivariate analysis derived from a randomized controlled trial examining risk factors for PIVC failure, also revealed significant associations between being female and the occurrence of phlebitis [76,80]. Various explanations have been proposed for these findings, including variations in hormonal levels and differences in fat tissue distribution [80]. Furthermore, women tend to have smaller vessel sizes than men, which may explain why women are more susceptible to developing phlebitis [80]. Additionally, women have been underrepresented in clinical studies evaluating medical devices, making it more challenging to understand the effectiveness of these devices for the female population [81]. This underrepresentation can affect our ability to draw conclusions about why more women experience phlebitis or complications related to PIVCs.

Regarding wards, there was an 81% increase in the odds of developing phlebitis in the observation unit. During our study, the observation unit underwent structural changes that involved many new employees, which may have led to a lack of guidance from experienced nurses to ensure good PIVC quality. Additionally, leadership may not have been sufficiently focused on quality improvement during this period of transition. Other studies have also found that adequate nursing staffing [81] and a ward culture with the presence of supportive leaders and expert nurses impact nurses’ PIVC care practice [10].

In our study, we implemented a care bundle featuring several interventions aimed at preventing complications related to PIVCs, which resulted in a decreased prevalence of phlebitis. Care bundles are designed to enhance patient treatment and should be evidence-based [5]. A systematic review revealed significant inconsistency in care bundles across previous studies, with not all bundles adhering to clinical guidelines [35]. Numerous patient injuries can be prevented by implementing enhanced patient safety measures [82], and the complications associated with PIVCs are most effectively mitigated through strict adherence to guideline recommendations [5]. Although evidence-based standardized bundles for PIVC care have been lacking [35], care bundles have increasingly attracted clinical attention [83]. Despite the numerous interventions developed to enhance the quality of PIVCs, there is a notable absence of implementation frameworks that could improve the effectiveness of these interventions [84]. In our study, we employed the Quality Improvement Model [47] as a framework, ensuring that the care bundles were based on clinical guidelines. However, implementing multiple interventions simultaneously may complicate the process of determining which specific interventions are truly effective [33]. This situation underscores the need for a standardized, evidence-based bundle for PIVC care [35], as well as the necessity for high-quality implementation studies in PIVC research [84].

### 5.3. Quality of the PIVC Dressing and IV Connection and Documentation

Significant improvements were observed in the condition of the dressings, particularly regarding issues such as dislodgement, contamination with blood or fluids, and the integrity of the edges, which were assessed for looseness or lifting. PIVC dressings are specifically designed to prevent complications and failures [85] by properly anchoring the PIVC with a sterile and transparent dressing [5]. Additionally, these dressings act as a microbial barrier [13], preventing skin bacteria from entering the patient’s bloodstream and causing infection [7]. A prospective observational study identified incorrect PIVC dressings as a predictor of phlebitis [72], highlighting the critical importance of securing the PIVC sufficiently. Although our study noted improvements in dressing care, many PIVCs remained in suboptimal conditions, which may expose patients to several complications. This situation underscores the ongoing need for awareness and education regarding optimal PIVC care, including appropriate dressings and IV connections.

Post-intervention, we found that 48% of the PIVCs had satisfactory documentation of their indication in the chart, and 55.1% had daily observation and maintenance recorded. These figures indicate a statistically significant improvement from the baseline. However, some residual gaps remain in the documentation domain. The overall mean indwell time for PIVCs was 1.88 days at baseline and 1.81 days post-intervention, but this difference was not statistically significant. Studies have shown that idle PIVCs may be associated with PIVC-BSIs [33] and that PIVCs inserted for more than three days increase the likelihood of PIVC colonization [1]. To mitigate the risk of infections associated with PIVCs, WHO recommends that all healthcare personnel receive training on the appropriate indications for their use [5]. Studies have identified a significant percentage of PIVCs as idle [8,9,10,12], often inserted in anticipation of potential emergencies [10]. Although we did not measure the number of idle PIVCs in our study, findings from other studies suggest that some may indeed be idle, as many of the PIVCs included in our study lacked documented indications for their use. In accordance with nursing standards, accurate documentation of PIVCs is essential for tracking potential complications and assisting healthcare personnel in maintaining PIVCs when necessary [13]. In our study, documentation of PIVCs in both the medical charts and on the dressings showed a statistically significant improvement. However, the documentation of daily observations, care, and indication assessments in the charts remained inadequate for nearly half of the PIVCs. This deficiency in documentation could ultimately lead to patient harm and increased healthcare costs.

### 5.4. PIVC-miniQ Sum Score

To the best of our knowledge, no other studies utilizing the PIVC-miniQ tool have compared the overall PIVC quality among different PIVC types. However, other studies that implemented closed integrated PIVCs have reported a reduction in complications or risks of complications [53,54,55,56,57,58]. Subsequently, three systematic reviews and meta-analyses were conducted [3,52,86]. Matthews et al. [3] and Gidaro et al. [52] found no significant difference in the incidence of phlebitis or the risk of developing phlebitis between closed integrated PIVCs and other PIVC designs. In our unadjusted regression analysis, we found that closed integrated PIVCs had 20% lower odds of developing phlebitis compared to ported PIVCs; however, this difference was not statistically significant. When comparing PIVC type and PIVC-miniQ sum score post-intervention, our study revealed a significant difference in the PIVC-miniQ sum score, indicating that closed integrated PIVCs exhibit overall better PIVC quality. Additionally, we observed a declining trend in the prevalence of phlebitis during the intervention period. Dobrescu et al. [86] found minimal to no differences in the rates of local infections and phlebitis when comparing PIVC types. However, this review excluded studies that utilized care bundles [86]. Furthermore, our study incorporated several interventions, including closed integrated PIVCs, suggesting that care bundles comprising multiple interventions are necessary to enhance PIVC quality [52].

Our study demonstrates a significant improvement in the PIVC-miniQ sum score following the intervention. To our knowledge, no other studies utilizing the PIVC-miniQ have compared PIVC quality before and after an intervention. The systematic review conducted by Ray-Barruel et al. [35] identified 22 distinct components of care bundles related to the insertion and maintenance of PIVCs, highlighting the range of interventions aimed at enhancing PIVC quality and reducing complications. Consequently, standardization of these bundle components is essential [35]. Nevertheless, our quality improvement study employed care bundle intervention recommendations from the NIPH guideline [24], and our results suggest that these interventions can improve the quality of PIVCs.

Høvik et al.’s [10] mixed-methods study revealed a gap in education regarding the maintenance of PIVCs beyond the insertion technique. It appears that PIVC maintenance education is often insufficient in many nursing programs, resulting in a reliance on individual practices and the prevailing culture surrounding PIVCs within each ward [10]. In contrast, our interventions included instruction on both the maintenance and insertion of PIVCs. These training sessions were delivered by the hospital’s infection prevention nurses and involved participation from all wards, ensuring consistent training across the entire hospital.

### 5.5. Implications for Practice

In response to our findings, we have implemented monthly evaluations of PIVCs and established feedback mechanisms for healthcare staff to foster ongoing awareness of continuous improvement in PIVC quality. Additionally, educational sessions on PIVC care and the prevention of complications are now conducted for newly hired nurses and students. The result of this study indicates that the quality of the closed integrated PIVCs is superior to that of the ported PIVCs for overall PIVC quality. This finding should encourage hospital leaders to consider implementing closed integrated PIVCs to enhance patient safety. Besides, a randomized controlled trial performing a cost-benefit analysis found that closed integrated systems achieved overall cost savings compared to open systems [57].

In this longitudinal quality improvement study, we demonstrate that the PIVC-miniQ tool [39] can successfully measure the local improvement of interventions designed to enhance PIVC quality. These findings offer valuable insights for future quality improvement studies aimed at preventing complications associated with PIVCs. Our study may also provide important insights into nurses’ care and practices regarding PIVCs, highlighting discrepancies between clinical guidelines and actual practice. This underscores the significance of continuous improvement efforts and the need to raise awareness among healthcare providers to ensure compliance with established guidelines.

## 6. Strengths and Limitations

The primary strength of this study was the longitudinal measurement of PIVC quality before and after the implementation of a care bundle. Additionally, the use of a reliable and validated instrument is a strength that ensures the accuracy of our findings [39]. Moreover, the team comprised a small group of experienced nurses with minimal turnover, specifically trained in screening PIVCs, ensuring consistency in data collection and contributing to the reliability of our findings [45]. Data collection was performed by pairs of nurses, allowing for consultation in case of uncertainty during the PIVC examination. Although this strengthens the reliability, including images of PIVC complications and performing inter-rater reliability testing among raters would have further improved the reliability [41]. However, earlier studies have found the ICC to be satisfactory [39,43,65]. Our unannounced observations may have increased nurses’ awareness of removing idle PIVCs or those with complications and ensuring proper documentation, potentially resulting in a positive outcome known as the Hawthorne effect [45].

The availability of a large dataset allowed us to conduct logistic regression analysis to identify predictors of phlebitis [87]. However, our measurements did not include precise PIVC indwell time, which could also serve as a potential predictor [72]. Phlebitis can result from various causes, including the infusion of vein-irritating medications [13,40]. Our study did not document specific medications administered beyond antibiotics, limiting our ability to analyze associations between medications and phlebitis occurrence. Further, we used a threshold of ≥1 as a criterion for defining phlebitis, which could have overestimated the prevalence. Confusion surrounding a phlebitis definition suggests that studies should report individual signs and symptoms, as done in Table 2 [14,88].

Our study did not record the number of patients in the wards, PIVCs per patient, or the number of patients without a PIVC. This limitation hinders our ability to assess potential improvement in the occurrence of idle PIVCs. Additionally, we lacked data on PIVC-related BSIs prior to the intervention, limiting our capacity to present comparable results. Another methodological limitation is our inability to determine which specific intervention or combination of interventions in the care bundle had the greatest impact on outcomes due to their simultaneous implementation.

This quality improvement study was conducted in one hospital, where the observed results are indicators of local improvements. Therefore, results from this study may not be generalizable to other settings. However, the study included patients from medical and surgical wards, as well as the intensive care unit, representing a diverse patient population. Benchmarking results with other hospitals can contribute to improving clinical practice [14]. Additionally, the increased use of the PIVC-miniQ may facilitate better comparison of PIVC quality between hospitals.

A limitation of this study is its pre–post design, with pre-measurements serving as a control group. A more robust approach would have been a randomized controlled trial (RCT) with a simultaneous control group, which would eliminate bias from treatment and patient changes over time, thus strengthening causal inference. An RCT would also facilitate continuous data collection and better management of time trends in both groups. Our data supports the feasibility of designing an RCT with sufficient power to detect a clinically significant effect. Post hoc power analyses are not recommended [89]. However, a new RCT designed with a significance level of 5% and power of 90% to detect a minimum clinical reduction of 33% in phlebitis (from 15% to 10%) would, par example, require at least n = 1836 in a two-sided test and n = 742 in a one-sided test. Another limitation was the lack of records for individuals with multiple PIVCs, which hindered our ability to account for dependency between the observations in the statistical tests and the regression analysis. This may have led to too significant *p*-values and narrow confidence intervals. Another potential weakness is the possible dependence between observations from the same ward, which could lead to deflated *p*-values. However, we believe this dependency is minimal, as adjusting for ward differences in our analyses slightly mitigates this bias. Lastly, a new documentation variable has not been evaluated for reliability and validity, which would be best addressed in a separate study. Furthermore, variability in phlebitis definitions, study designs, contexts, care bundle components, and endpoint measures complicates comparisons between studies [90].

## 7. Conclusions

To the best of our knowledge, this is the first study to conduct a longitudinal quality improvement initiative evaluating local improvements of a care bundle designed to minimize complications associated with PIVCs using the PIVC-miniQ tool. Our results indicate that this care bundle, comprising several interventions, improves PIVC quality and reduces the prevalence of phlebitis. The study also highlights the importance of observation, care, and documentation of PIVCs in reducing complications. Despite the availability of various care bundles, no specific one has been proven highly effective. Further research is needed to identify and develop the most effective strategies for preventing complications related to PIVCs in more robust research designs.

## Figures and Tables

**Table 1:** Descriptive characteristics for 1330 PIVCs at a hospital in Norway conducted in June and August 2022 (pre-intervention) and from November 2022 to November 2023 (post-intervention).

	Pre-Intervention (n = 185)			Post-Intervention (n = 1145)			Total (n = 1330)		Difference Pre-Post-Intervention		
Variables									Mean	(SD)	*p*-Value
Age (years), n, mean (SD)	185	71.6	(16.8)	1145	70.2	(16.8)	1330		1.5	(1.3)	0.266 ^a^
Gender, n (%)											0.164 ^b^
Female	79	(42.7)		552	(48.2)		631	(47.4)			
Male	106	(57.3)		593	(51.8)		699	(52.6)			
PIVC indwell days, n, mean (SD)	172	1.88	(1.3)	1110	1.81	(1.7)	1282		0.08	(0.1)	0.506 ^a^
PIVC site, n (%)											0.201 ^b^
Hand	38	(20.5)		269	(23.5)		307	(23.1)			
Wrist	11	(5.9)		84	(7.3)		95	(7.1)			
Forearm	86	(46.5)		475	(41.5)		561	(42.2)			
Antecubital fossa	39	(21.1)		284	(24.8)		323	(24.3)			
Foot	8	(4.3)		20	(1.7)		28	(2.1)			
Upper arm	3	(1.6)		12	(1.0)		15	(1.1)			
Chest	0	(0.0)		1	(0.1)		1	(0.1)			
PIVC size (gauge), n (%)											0.405 ^b^
22 G (blue)	33	(17.8)		192	(16.8)		225	(16.9)			
20 G (pink)	96	(51.9)		515	(45.0)		611	(45.9)			
18 G (green)	55	(29.7)		427	(37.3)		482	(36.2)			
16 G (grey)	1	(0.5)		3	(0.3)		4	(0.3)			
Unknown				8	(0.6)		8	(0.6)			
Insertion environment, n (%)											<0.001 ^b^
Ambulance/EMS	9	(4.9)		50	(4.4)		59	(4.4)			
Emergency department	73	(39.5)		434	(37.9)		507	(38.1)			
Operating theatre	19	(10.3)		170	(14.8)		189	(14.2)			
Hospital ward/unit/ICU	71	(38.4)		431	(37.7)		502	(37.7)			
Radiology/procedure room	1	(0.5)		1	(0.1)		2	(0.2)			
Unknown	12	(6.5)		59	(5.2)		71	(5.3)			
Ward, n (%)											0.010 ^b^
Surgical ward	40	(21.6)		275	(24.0)		315	(23.7)			
Medical ward 2	60	(32.4)		289	(25.2)		349	(26.2)			
Medical ward 1	58	(31.4)		296	(25.9)		354	(26.6)			
Intensive care unit	12	(6.5)		99	(8.6)		111	(8.3)			
Observation unit	15	(8.1)		186	(16.2)		201	(15.1)			
PIVC, n (%)											<0.001 ^b^
Closed integrated PIVC	0	(0.0)		497	(43.4)		497	(37.4)			
Ported PIVC	185	(100.0)		634	(55.4)		819	(61.6)			
Non ported PIVC	0	(0.0)		14	(1.2)		14	(1.1)			
Antibiotics, n (%)											0.523 ^b^
Yes	59	(31.9)		372	(32.5)		431	(32.4)			
No	106	(57.3)		747	(65.2)		853	(64.1)			
Unknown	20	(10.8)		26	(2.3)		46	(3.5)			

Abbreviations: PIVC = peripheral intravenous catheter; G = gauge: blue 22 G (0.9 mm), pink 20 G (1.1 mm), green 18 G (1.3 mm), grey 16 G (1.7 mm); ICU = intensive care unit; EMS = emergency medical services; SD = standard deviation; ^a^ independent *t*-test with separate variances; ^b^ chi-square test.

**Table 2:** Descriptive results and comparisons for each item on the PIVC-miniQ for 1330 PIVCs at a hospital in Norway conducted in June and August 2022 (pre-intervention) and from November 2022 to November 2023 (post-intervention).

PIVC-miniQ Domain	Pre-Intervention (n = 185)		Post-Intervention (n = 1145)		Total (n = 1330)		Chi-Square Test
Items	n	%	n	%	n	%	*p*-Value
**Phlebitis-related signs and symptoms**							
Pain	20	10.8	73	6.4	93	7.0	0.028
Redness > 1 cm from insertion site	9	4.9	30	2.6	39	2.9	0.093
Swelling	5	2.7	29	2.5	34	2.6	0.892
Warmth at insertion site	0	0.0	0	0.0	0	0.0	1.000
Purulence	0	0.0	0	0.0	0	0.0	1.000
Streak/red line along the vein	0	0.0	0	0.0	0	0.0	1.000
Induration, hardness of tissue > 1 cm from insertion site	7	3.8	10	0.9	17	1.3	<0.001
Palpable hard vein beyond tip	0	0.0	0	0.0	0	0.0	1.000
**PIVC dressing and IV connection**							
Partial/complete dislodgement	1	0.5	2	0.2	3	0.2	0.330
Soiled with blood or fluids	81	43.8	289	25.2	370	27.8	<0.001
Loose or lifting dressing edges	40	21.6	168	14.7	208	15.6	0.016
Sterile dressing missing	0	0.0	4	0.4	4	0.3	0.421
Blood in line	73	39.5	216	18.9	289	21.7	<0.001
**Documentation**							
Insertion date not documented on PIVC dressing	124	67.0	511	44.6	635	47.7	<0.001
Documentation of indication in chart is missing	185	100.0	595	52.0	780	58.7	<0.001
PIVC insertion date in chart is missing	20	10.8	91	8.0	111	8.4	0.191
Daily documentation of observation, care, and indication assessment in chart is missing	119	64.3	514	44.9	633	47.6	<0.001

Abbreviations: PIVC = peripheral intravenous catheter; PIVC-miniQ = Peripheral Intravenous Catheter-Mini Questionnaire; IV = intravenous.

**Table 3:** Results from logistic regression analyses of the PIVC-miniQ domains phlebitis, PIVC dressing, and IV connection and documentation, with a sum score of at least equal to 1 for 1330 PIVCs at a hospital in Norway, conducted in June and August 2022 (pre-intervention) and from November 2022 to November 2023 (post-intervention).

PIVC-miniQ	Pre-Intervention (n = 185)		Post-Intervention (n = 1145)		Total (n = 1330)		Chi-Square Test	
Domains	n	%	n	%	n	%	*p*-Value	RR
Phlebitis	28	15.1	108	9.4	136	10.2	0.018	1.61
PIVC dressing and IV connection	130	70.3	530	46.3	660	49.6	<0.001	1.52
Documentation	185	100.0	901	78.7	1086	81.7	<0.001	1.27

Abbreviations: PIVC = peripheral intravenous catheter; IV = intravenous; PIVC-miniQ = Peripheral Intravenous Catheter-Mini Questionnaire; RR = risk ratio.

**Table 4:** Results from logistic regression analyses of phlebitis with respect to potential predictors for 1330 PIVCs at a hospital in Norway during 2022–2023.

	Unadjusted Models					Fully Adjusted Model (n = 1276)			Final Model (n = 1276)		
Variable (Predictors)	n	OR	95% CI	*p*-Value	VIF	OR	95% CI	*p*-Value	OR	95% CI	*p*-Value
Intervention	1330	0.58	(0.37, 0.91)	0.024	1.0	0.51	(0.30, 0.87)	0.016	0.53	(0.33, 0.86)	0.014
Ward	1330			<0.001				<0.001			<0.001
Medical ward 1	354	1.00	(Reference)		-	1.00	(Reference)		1.00	(Reference)	
Surgical ward	315	1.74	(1.05, 2.89)		1.6	1.84	(0.98, 3.45)		1.63	(0.96, 2.76)	
Medical ward 2	349	1.51	(0.91, 2.50)		1.5	1.72	(1.00, 2.95)		1.56	(0.92, 2.64)	
Intensive care unit	111	0.00	cc		cc	0.00	cc		0.00	cc	
Observation unit	201	1.81	(1.03, 3.16)		1.3	1.66	(0.92, 3.01)		1.75	(0.98, 3.13)	
Gender	1330			0.003				0.006			0.004
Male	699	1.00	(Reference)		-	1.00	(Reference)		1.00	(Reference)	
Female	631	1.72	(1.20, 2.47)		1.0	1.70	(1.16, 2.50)		1.75	(1.20, 2.55)	
Insertion site	1330			0.203				0.700			
Forearm	561	1.00	(Reference)		-	1.00	(Reference)				
Antecubital fossa	323	1.30	(0.84, 2.00)		1.0	1.13	(0.68, 1.86)				
Chest	1	0.00	cc		1.2	0.00	cc				
Foot	28	0.00	cc		1.8	0.00	cc				
Hand	307	1.07	(0.68, 1.70)		1.3	1.16	(0.70, 1.90)				
Wrist	95	0.85	(0.39, 1.84)		1.1	0.82	(0.37, 1.83)				
Upper arm	15	0.66	(0.09, 5.09)		1.8	1.02	(0.12, 8.81)				
PIVC gauge (mm)	1322	3.13	(0.90, 10.90)	0.071	1.4	4.62	(0.92, 23.13)	0.060	5.42	(1.36, 21.56)	0.016
PIVC type	1330			0.107				0.544			
Ported PIVC	819	1.00	(Reference)		-	1.00	(Reference)				
Closed integrated PIVC	497	0.80	(0.55, 1.16)		1.1	1.06	(0.67, 1.69)				
Non ported PIVC	14	0.00	cc		-	0.00	cc				
Insertion environment	1330			0.287				0.345			
Emergency department	507	1.00	(Reference)		-	1.00	(Reference)				
Operating theatre	189	0.97	(0.57, 1.67)		1.1	0.80	(0.38, 1.72)				
Ambulance/EMS	59	1.88	(0.92, 3.84)		1.0	1.74	(0.80, 3.79)				
Radiology/procedure room	2	0.00	cc		1.1	0.00	cc				
Hospital ward/unit/ICU	502	0.81	(0.53, 1.23)			0.97	(0.62, 1.51)				
Unknown	71	0.62	(0.24, 1.61)			0.48	(0.16, 1.39)				
Antibiotics	1284	0.62	(0.41, 0.94)	0.020		0.77	(0.49, 1.21)	0.246			

Abbreviations: PIVC = peripheral intravenous catheter; OR = odds ratio; CI = confidence interval; *p*-value = from likelihood ratio test; ICU = intensive care unit; EMS = emergency medical service; cc = cannot be calculated, VIF = variance inflation factor.

## Data Availability

The data presented in this study are available on request from the corresponding author. The data are not publicly available due to privacy and ethical restrictions.

## Supplementary Materials

The following supporting information can be downloaded at: https://www.mdpi.com/article/10.3390/nursrep15110379/s1.

## Public Involvement Statement

No public involvement in any aspect of this research.

## Guidelines and Standards Statement

This study is reported in accordance with the recommendations in the Standards for Quality Improvement Reporting Excellence 2.0 guidelines [46].

## Abbreviations

The following abbreviations are used in this manuscript:

PIVC	Peripheral Intravenous Catheter
IV	Intravenous
BSI	Bloodstream Infection
*S. aureus*	*Staphylococcus aureus*
HAI	Healthcare-Associated Infection
WHO	World Health Organization
PIVC-miniQ	The Peripheral Intravenous Catheter Mini-Questionnaire
NIPH	Norwegian Institute of Public Health
SQUIRE 2.0	Standards for Quality Improvement Reporting Excellence 2.0
ICC	Intraclass Correlation Coefficient
SD	Standard Deviation
REK	The Regional Committee for Medical and Health Research Ethics
G	Gauge
ICU	Intensive Care Unit
EMS	Emergency Medical Services
RR	Risk Ratio
PIVC mm	Peripheral Intravenous Catheter millimeters
OR	Odds Ratio
CI	Confidence Interval
CC	Cannot Be Calculated
i.e.	That Is
