# Supplementary material for: Implementation of an Infection Prevention Care Bundle for Peripheral Intravenous Catheters (PIVCs): A Quality Improvement Study to Enhance PIVC Quality and Reduce Complications
Source: Nurs Rep. 2025 Oct 24;15(11):379. doi: 10.3390/nursrep15110379 (PMC12655417; doi:10.3390/nursrep15110379)
Supplement: Supplementary file 1 [file nursrep-15-00379-s001.zip › nursrep-3878893-supplementary.pdf]

PIVC-miniQ

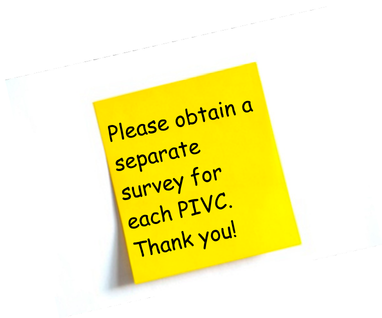

Hospital/Site	
Ward/Unit	
Antibiotics	□ Yes □ No
PIVC nr.	
Age of patient	
Gender of patient	□ Male □ Female
Date of PIVC insertion	
Date of review	

**PIVC position/site:**<br>**Right** □ **Left** □	**Catheter Gauge PIVC size:**	**PIVC type:**	**Where was the catheter inserted?**<br>*(ask patient if not documented)*
□ Hand<br>□ Wrist<br>□ Forearm<br>□ Antecubital fossa<br>□ Foot<br>□ Head/neck<br>□ Upper arm	□ 24 G Yellow<br>□ 22 G Blue<br>□ 20 G Pink<br>□ 18 G Green<br>□ 16 G Grey<br>□ 14 G Brown/Orange	□ Ported PIVC<br>□ Closed integrated PIVC<br>□ Non ported PIVC	□ Ambulance/ Emergency Medical Services<br>□ Emergency department<br>□ Operating room<br>□ General ward/ unit/ clinic/ Intensive Care Unit<br>□ Radiology/ Procedure room<br>□ Unknown

**PIVC site assessment:**			**Iv dressing and iv connection assessment:**		
Pain/tenderness on palpation	□ Yes	□ No	Soiled with blood or fluids	□ Yes	□ No
Redness > 1 cm from insertion site	□ Yes	□ No	Loose or lifting edges	□ Yes	□ No
Swelling > 1 cm from insertion site	□ Yes	□ No	Tape only	□ Yes	□ No
Warmth	□ Yes	□ No	Blood in line	□ Yes	□ No
Purulence	□ Yes	□ No	PIVC insertion date not documented on dressing	□ Yes	□ No
Streak/ red line along vein	□ Yes	□ No			
Induration/ hardness of tissues>1 cm	□ Yes	□ No			
Palpable hard vein beyond IV tip	□ Yes	□ No			
Partial/ complete dislodgement PIVC	□ Yes	□ No			

**Indication:**	**Documentation:**
□ Indication unknown	Date of PIVC insertion in patient chart is lacking □ Yes □ No<br><br>Daily documentation of observation, care and indication assessment in chart is missing □ Yes □ No

Developed by St.Olav´s Hospital

Haraldsplass
Diakonale sykehus
